# A Decentralized Wireless Solution to Monitor and Diagnose PV Solar Module Performance Based on Symmetrized-Shifted Gompertz Functions

**DOI:** 10.3390/s150818459

**Published:** 2015-07-29

**Authors:** Angel Molina-García, José Carlos Campelo, Sara Blanc, Juan José Serrano, Tania García-Sánchez, María C. Bueso

**Affiliations:** 1 Department of Electrical Engineering, Universidad Politécnica de Cartagena, 30202 Cartagena, Spain; 2 Institute ITACA, Universitat Politècnica de València, 46071 Valencia, Spain; E-Mails: jcampelo@disca.upv.es (J.C.C.); sablacla@disca.upv.es (S.B.); jserrano@disca.upv.es (J.J.S.); 3 Renewable Energy Institute DEEEAC, Universidad de Castilla La-Mancha, 02071 Albacete, Spain; E-Mail: tania.garcia@uclm.es; 4 Department of Applied Mathematics and Statistics, Universidad Politécnica de Cartagena, 30202 Cartagena, Spain; E-Mail: mcarmen.bueso@upct.es

**Keywords:** monitoring, photovoltaic power systems, solar power generation, wireless sensor network

## Abstract

This paper proposes and assesses an integrated solution to monitor and diagnose photovoltaic (PV) solar modules based on a decentralized wireless sensor acquisition system. Both DC electrical variables and environmental data are collected at PV module level using low-cost and high-energy efficiency node sensors. Data is real-time processed locally and compared with expected PV module performances obtained by a PV module model based on symmetrized-shifted Gompertz functions (as previously developed and assessed by the authors). Sensor nodes send data to a centralized sink-computing module using a multi-hop wireless sensor network architecture. Such integration thus provides extensive analysis of PV installations, and avoids off-line tests or post-processing processes. In comparison with previous approaches, this solution is enhanced with a low-cost system and non-critical performance constraints, and it is suitable for extensive deployment in PV power plants. Moreover, it is easily implemented in existing PV installations, since no additional wiring is required. The system has been implemented and assessed in a Spanish PV power plant connected to the grid. Results and estimations of PV module performances are also included in the paper.

## 1. Introduction

During the last decade, governments and public organizations have encouraged the integration of renewable energy resources into power systems, in an effort to decrease dependence on traditional fossil energy sources. These measures have been combined with pollutant emission reduction agreements. In total, 385 GW of new power capacity has been installed in the EU since 2000. Of this new power generation, over 55% was renewable and 92% renewable and gas combined. The net growth since 2000 of gas power (131.7 GW), wind (115.4 GW), and solar PV (photovoltaic) (80 GW) was at the expense of fuel oil (down 28.7 GW), coal (down 19 GW), and nuclear (down 9.5 GW) [1]. With the development of distributed generation systems, renewable electricity from PV sources became an energy resource in great demand [2]. The other renewable technologies (hydro, biomass, waste, CSP, geothermal, and ocean energies) have also been increasing their installed capacity over the past 13 years, albeit more slowly than wind and solar PV. Solar PV has thus experienced the most growth in recent years, close to 60% in Europe [3]. Recent studies estimate a 15% penetration of the PV market in Europe by 2030 [4]. However, connecting hundreds and thousands of renewable resources to the utility network introduces different dynamics in the system [5], and thus the distributed sources should be properly controlled to avoid unstable situations and failures. Actually, as the penetration level is continuously growing, controllability of active power and reactive power supplied by PV installations must be ensured in future systems [6]. As an attempt to minimize undesirable power oscillations and detect possible operational problems, an adequate monitoring system is usually proposed in the specific literature as a solution that produces a higher final energy yield than would be possible without monitoring [7]. Various data acquisition systems have been developed in recent years in the specific literature. In [8], a preliminary low-cost current-voltage (*I*-*V*) measuring system is presented, including results corresponding to seven crystalline Silicon PV modules. This system uses an array of resistors as load for the module, which are switched in and out of the circuit in such a way to enable the module *I*-*V* characteristics. In [9], I/O modular devices using *LabView* and transmission to a PC through serial port RS-232 are described. Estimations based on inferential statistics using the energy output of inverters can be found in [10], where the proposed methodology is assessed using PV installations with 132 modules. In [11], Power Line Communication (PLC) was proposed to perform a full monitoring tool. In this work, wireless communication was not selected since, according to the authors, the viability of the proposal was unclear, and then, its utilization was rejected for economic reasons. A more recent contribution also based on PLC can be found in [12], where losses are estimated of between 20% and 30% of the optimum plant production (as defined by the standard operating conditions) and the monitoring and control of PV systems is proposed as a reasonable solution to minimize production losses during the lifetime of the systems. However, PLC solutions usually present drawbacks as a consequence of DC signal compatibility requirements. A commercial data acquisition system consists of a Sunny Boy 1700 inverter, Sunny SensorBox and Sunny WebBox, tested to monitor a 1.72 kWp photovoltaic system installed on a flat roof [13]. The SensorBox and inverter are connected to the Sunny WebBox via a serial RS485 link and a Power Injector. A recent outdoor experimental laboratory for estimating the performance of PV plants in operating conditions is presented in [14]. This is a wired solution assessed with different PV modules. Nevertheless, the extension of this approach in other PV installations presents some drawbacks and maintenance requirements due to the necessity of additional wiring.

In the past few years, Wireless Sensor Networks (WSNs) have evolved considerably, providing low-cost and low-energy solutions with reliable data acquisition and real-time performance, ensuring loss-free transmission to the final destination. WSNs are becoming increasingly suitable solutions in various fields. Systems based on WSN communications are the subject of important research using prototypes and simulation models, including leveraging overhead, efficient spatial use with non-over-lapping nodes, and reliable data transport [15]. A wireless connection makes the network installation and physical maintenance easier, thereby providing portable solutions for reusable monitoring systems. In this framework, PV production plants are currently demanding real time monitoring with local and accurate information processing [16]. WSN could thus provide an extensive solution that integrates heterogeneous sensors, ranging from meteorological sensors and digital data acquisition to complex analogue signal processing. Indeed [17], discusses a preliminary application of WSN for monitoring PV installations. A gathering process is proposed that excludes the estimation of the PV module performance. A recent WSN approach can be found in [18], where the monitoring of a PV system at the panel level is presented with the aim of detecting the causes of efficiency losses. A single-diode-based equivalent circuit model, characterized with five parameters, is considered. An analytical method is used, including constraints during the minimization process, to estimate the five parameters.

With the aim of providing an integrated system to monitor and diagnosis PV installations at module-level, this paper describes and assesses a solution based on WSN low-cost architecture at PV module-level. The IEEE 802.15.4 open standard [19] is selected because of its remarkable characteristics in terms of reliability, cost-effectiveness, and low-power consumption. A multi-hop WSN architecture is proven to be feasible and effective for this application [20]. The main improvements with respect to similar contributions are the use of low-cost hardware, minimal power consumption, data transmission based on multi-hop communication, as well as the inclusion of a PV module model able to estimate the *I*-*V* solar module curve by using a non-implicit function with only three-parameters. These parameters can be determined for any solar irradiation and temperature values, avoiding minimizing processes and convergence problems and reducing considerably computational time costs and hardware requirements. Indeed, our proposal is in line with recent contributions for monitoring PV systems, which call for a reliable fault detection, an accurate localization of faulty components and an easy installation and commissioning [21]. Preliminary results focused on assessing the proposed WSN architecture can be found in [22].

The rest of the paper is organized as follows: Section 2 gives a brief review of the PV module modeling, and formally introduces the proposed function implemented at the sensor node level. Section 3 presents the global solution, structure and communication characteristics. Hardware components are described in Section 4. Results are discussed in Section 5 and, finally, some concluding remarks are found in Section 6.

## 2. PV Module Modeling: Symmetrized Shifted Gompertz Functions

The accuracy of PV plant simulations is strongly dependent on the PV cell modeling [23]. As a consequence, a relevant number of contributions have focused on both PV cell and module modeling. Traditionally, the PV module equivalent model has been obtained from a set of individual PV cells connected in series (assumed to be identical in both direct and reverse bias behavior). Most contributions consider the *I*-*V* characteristic as a non-linear function, mainly dependent on a set of variables, including solar irradiation and temperature (G & T), and using an equivalent electrical circuit [24]. Under these assumptions, two different topologies have been mainly proposed in the specific literature: the Double-Diode Model (DDM) [23] and Single-Diode Model (SDM) [25,26]. Both approaches are usually described by implicit expressions, involving a remarkable number of parameters to be estimated. Various iterative processes have been recently proposed with this aim in the specific literature [27,28]. Regarding efficiency/power correlations, an extensive analysis of PV installation electric performance with the operating temperature can be found in [29].

The integration of these previous solutions in practical applications presents important limitations and possible convergence problems for correctly estimating *I*-*V* curves, especially when hardware limitations and computational time cost constraints are required. To overcome these drawbacks, a three-parameter PV module model has been recently proposed and assessed by the authors [30]. The suggested three-parameter model to characterize an *I*-*V* curve from G & T values is based on a non-implicit Symmetrized Shifted Gompertz (SSG) function given by the following expression,

(1) $$I=k\cdot e^{-e^{b(V-\gamma )}}-(k/e),\;0\leq V\leq \gamma$$

where *k*, γ and *b* are the three parameters corresponding to the short-circuit current (*I_sc_*) and the open-circuit voltage (*V_oc_*) given by the expressions:

(2) $$I_{sc}=k\cdot e^{-e^{-b\cdot \text{γ}}}-(k/e)$$

(3) $$V_{oc}=\text{γ}$$

**Figure 1:**
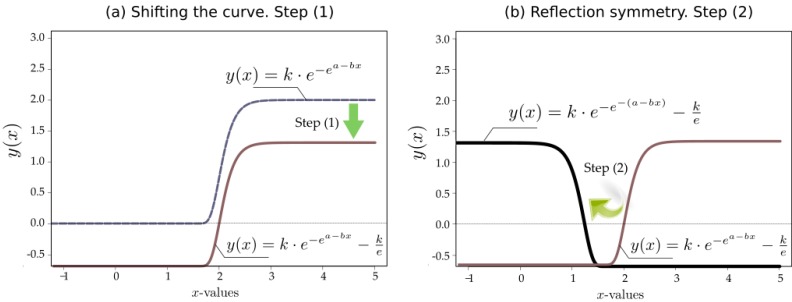
Symmetrized Shifted Gompertz function. Graphical deduction. Shifting the initial curve (**a**) and application of reflection symmetry (**b**).

The process is illustrated in Figure 1, where steps—(1) and (2) in the figure—can be visualized. Specifically, starting from a standard Gompertz curve $y\left(x\right)=ke^{-e^{a-b\cdot x}}$ and following steps: (1) shifting the curve so that it hits the x-axis at the value *a/b*; (2) applying to the result a reflection symmetry, with respect to the vertical line *x = a/b* and thus, obtaining the curve given by Equation (1), where

(4) γ=a/b

The estimation of γ, *b*, and *k* can be easily performed by using data typically provided by the manufacturers: *I_sc_*, *V_oc_*, and the maximum power point (*V_MPP_*, *I_MPP_*), which correspond to three points on the curve. The adjusted *I*-*V* curve is thus a non-implicit function with only three parameters to be fitted and then significantly decreases the computational time costs as well as the convergence problems of previous nonlinear approaches. The proposed model and the corresponding fitted parameters have been implemented in a reduced low-cost hardware solution discussed in Section 3. The estimation of parameters has been implemented using R-language [31]. This non-implicit PV module model gives a proper estimation of the PV module performance in real-time, by comparing collected DC data with the *I*-*V* characteristic estimated by the Gompertz approach. The proposed model has been assessed with non-silicon thin-film and c-Si PV modules, showing suitable results under both different technologies. Indeed, and only considering as input data both G & T values, it is possible to provide the entire *I*-*V* curve with neglected computational time cost. From this curve, measured DC values can be compared with the computed MPP and the *I*-*V* curve, given additional information not only in terms of efficiency but also as a measure for the design, installation, and maintenance of the PV systems. Actually, [32] affirms that only the experimental measurement of the *I*-*V* curve allows us to know with precision the electrical parameters of a PV cell. Moreover, [33] reports module performance results after 11 years of field exposure including not only MPP variations, but also the decreasing presented by average module short-circuit current.

## 3. The Proposed Wireless Solution: General Overview

The proposed solution consists of a set of sensor nodes that collect instantaneous DC-voltage and DC-current values at the PV module level, as well as temperature and solar radiation parameters. According to the implemented SSG function, estimations of expected DC values are also determined by these nodes. Both electrical and environmental values are then transmitted from these sensor nodes to a sink node by means of a type of hop through an 802.15.4 network. Consequently, a distributed and decentralized system is proposed, where each sensor node can be considered as a secondary node, the primary nodes being a set of sink nodes operating in complete autonomy.

**Figure 2:**
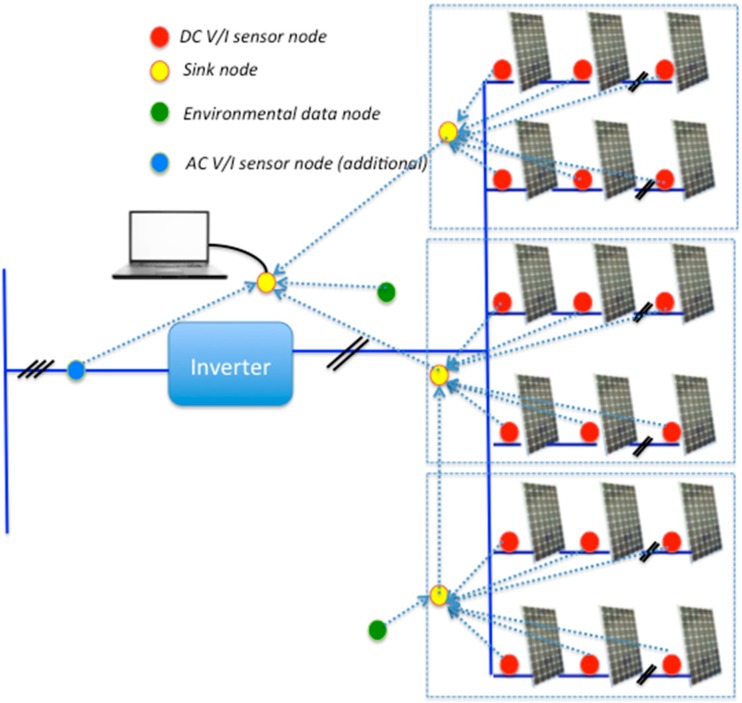
General scheme of the proposed wireless solution.

The sink nodes can be connected to an external computing system with the aim of extending the capacity of storing data or providing additional analysis. The sink node is instrumented to Ethernet, USB, and RS-232, offering real-time accessibility, remote connections, and database storage. These characteristics provide a remarkable extension of the proposed system, in terms of avoiding the local presence in PV power plants as well as off-line checking and disconnections of PV modules. The measuring and gathering process is carried out by the sensor nodes with a configurable sample time. The configuration of the proposed solution is highly flexible, depending on the number of PV modules, strings, arrays, and the layout of the PV installation. Figure 2 shows an example where each sink node is able to gather data from different arrays and send to the other sink nodes.

An overview focused on current PV system pricing trends can be found in [34]. According to this report, averaged installed prices for PV systems were estimated between 3.3$/W_DC_ and 1.8$/W_DC_ in 2013. By considering these estimations as well as common nominal power values for PV modules, the proposed wireless sensor solution supposes a low-cost system. In fact, for mass production series large enough to achieve economies of scale, sensor nodes cost approximately between 11 and 13$/unit by considering orders over 5000 units. This cost can account between 5 and 7 percent of the previous $/W_DC_ values. The initial investment is amortized after a short-term period, providing a complete monitoring system at the PV module level. In addition, it can also be possible to integrate the wireless sensor nodes within the PV solar module during the manufacturing process, bringing the cost of the proposed solution even lower.

Communication between sensor nodes and a sink node is implementing by following a *star-topology*. Communication and exchange of information between sink nodes is implemented by a multi-hop strategy to give a suitable solution for long distances. Actually, the applicability of single-hop WSNs is limited, since multi-hop WSNs are being deployed even in current industrial scenarios [35]. Subsequently, an 802.15.4 network is selected and implemented as a logical tree, in which each sink node fulfills message router functions to offer multi-hop communication. Additionally, 802.15.4 networks can be spread over the same set of customers using different network identifiers and/or different physical channels (at 2.4 GHz, sixteen different channels are available). A message sent by a sink sensor node is then routed to its father, and the father node to the corresponding father until achieving the end-sink node. The way a sink sensor node identifies the address of its father node is based on a dynamic configuration of the network developed in this work and described as follows: each sink node asks for a father node by broadcasting a request message. As several sinks can answer this request, according to a function of the radio signal quality and distance (in hops), each sink sensor node selects a father and sends a confirmation message.

**Figure 3:**
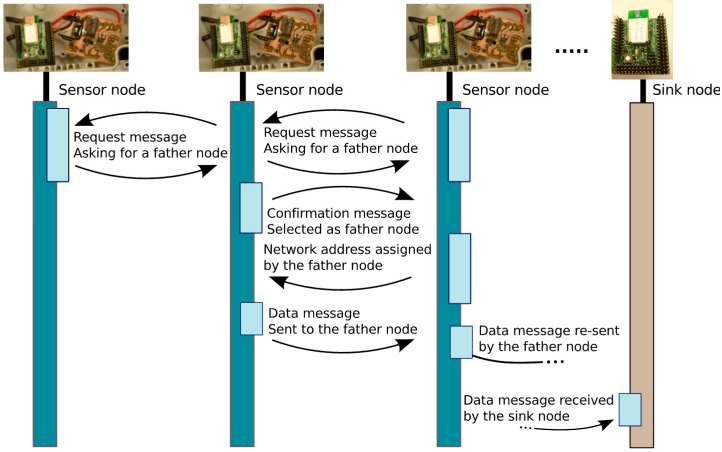
Multi-hop communication through sensor and sink nodes.

A network address is then assigned by the father following the addressing mechanism described in [36]. This identification system enables an easy integration of new sink nodes without any additional cost. Figure 3 schematically shows how the data is sent from a generic sensor node to the end sink node connected to a PC. There are alternatives to the synchronous network design in which nodes agree on their transmission periods based on an internal clock. It is possible to use additional hardware that enables the node to remain in low-power mode until a radio wake-up tone or beacon is detected. Some wake-on-radio devices reuse the radio transmission system to send the wake-on signal. This solution has the advantage of lower costs. The reception circuit enables all nodes to awaken a sleeping node. The design of a network with nodes equipped with this solution avoids the problem of agreeing synchronous sleep times at the expense of increased cost and energy consumption. Our system incorporates this asynchronous wake-up system [37] and drains 3 µA.

Relevant information regarding electrical performance of PV modules is provided by the sensor network in terms, for example, of predictive maintenance purposes. Electrical and environmental data collected by the sensor nodes and received by the end-sink node can be structured as a SQL database and remotely accessed from authorized clients by using different communication networks. Indeed, a software application has also been developed by the authors to give users friendly and complete on-line access to the monitored PV power plants.

## 4. Hardware Description

### 4.1. Sink Node

**Figure 4:**
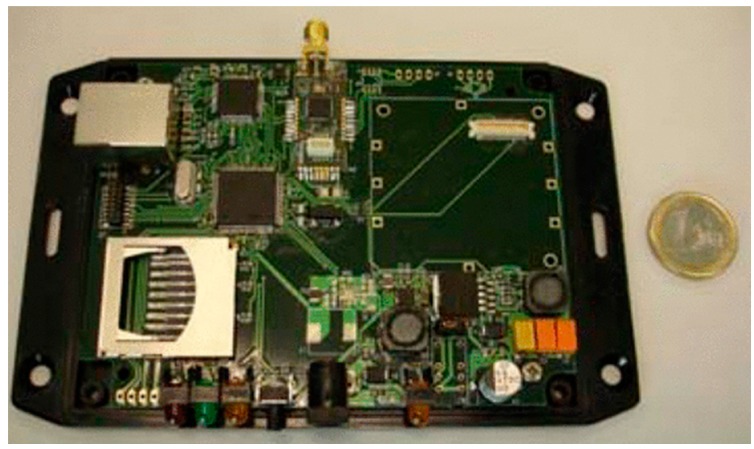
Sink node. General overview.

Sink nodes are implemented by means of two high performance microcontrollers, see Figure 4. A microcontroller is in charge of collecting data received from the network. This data is processed and can be sent to an external PC. The selected microcontroller is the STR912FAW44X developed by ST microelectronics running at 25 MHz, 97 kB of RAM memory, 544 kB of FLASH memory, digital input/output ports, serial ports, CAN, USB, and Ethernet [38]. A *NXP-Jennic* module has been connected to the TR912FAW44X to provide wireless interface based on 802.15.4 standard. The latest generations of *NXP-Jennic* modules are ultra-low-power, low-cost wireless microcontroller for wireless sensor networking applications based on the IEEE 802.15.4 standard, including *JenNet*, *JenNet-IP*, and *ZigBee* PRO applications [39]. These modules have an enhanced 32-bit RISC processor featuring improved coding efficiency and up to 32-MIPs performance. In addition, a fully compliant 2.4 GHz IEEE 802.15.4 transceiver, 128 kB of ROM and 128 kB of RAM are included to support a networking protocol stack and on-chip user applications, as well as a rich mix of user peripherals.

### 4.2. Sensor Node

These sensor nodes are in charge of collecting DC values (voltage and current) from the PV modules as well as estimating these electrical variables from the implemented SSG function and the collected G & T parameters. The DC voltage monitoring can be performed by means of a voltage divider. This solution has been adopted as an initial prototype. Nevertheless, safety issues in PV installations require isolated electronic subsystems to make independent signal and power supply [40,41]. Voltage monitoring can be thus implemented by using an optocoupler. In our case, a high reliability optocoupler, SFH615 from VISHAY [42], has been selected to isolate signal and power supply. This optocoupler provides an isolation voltage of 5.3 kV_RMS_ [43].

**Figure 5:**
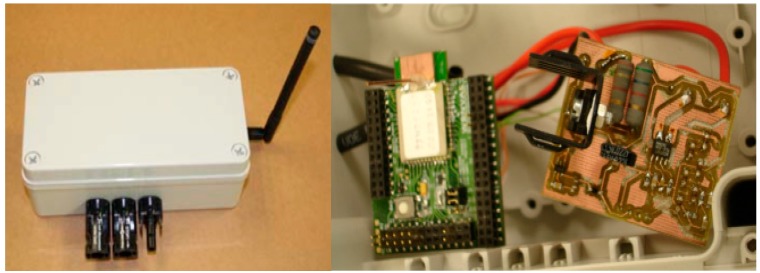
Sensor node. DC-voltage and current measurements.

The DC current monitoring process is performed by the Allegro ACS712 sensor [44], see Figure 5, and this provides a low-cost and accurate solution for AC/DC current measurements in industrial, commercial, and communication systems. This sensor is a Hall effect-based linear current Sensors IC, with 2.1 kV_RMS_ isolation and a low resistance current conductor [44]. The device consists of a precise, low-offset, linear Hall circuit with a copper conduction path located near the surface of the die. The current flowing through this copper conduction path generates a magnetic field that the Hall IC converts into a proportional voltage. Isolation between the current PV line and the wireless measuring node is thus obtained. The series configuration of PV modules at string level allows us to remove current sensors corresponding to the same DC current value. It would be then possible to decrease the cost of the sensor nodes, taking into account that the Allegro ACS712 sensor costs lower than €1.76 in orders over 5000 units. Nevertheless, a new application of the sensor nodes is currently under patent-pending status, which needs the inclusion of the DC current sensor in all nodes. For this reason, the Allegro ACS712 sensor has been considered by default in our sensor node prototype-board.

Voltage and current values are monitored every minute during the day with transmission frequency reduced at night. An additional module has also been integrated to provide advanced features for environmental measurement. In our case, a temperature probe sensor (PT1000) [45] with (−10, 80 °C) temperature range and a pyranometer in the range of (0, 100 mV) have been included as well. Platinum resistance thermometer (PT) is the industry standard method for accurate measurement of temperature in a wide temperature range. The pyranometer used in this application is the CMP3 model from Kipp & Zonen [46]. It is a thermopile sensor that measures the solar energy received from the total solar spectrum (W/m^2^) and the whole hemisphere, 180° field of view. It is a low-cost radiometer for accurate routine measurements. Furthermore, it has a robust 4 mm thick glass dome to protect the thermopile from external influences. The small size and sealed construction make this instrument an ideal choice for horticulture, monitoring solar energy installations, and industrial applications. This additional module can be adapted to batteries or energy harvesting in an IP 66 package. The sensor node performs average pre-processing and collecting processes. A circuit based on operational amplifiers provides the node with two sensor channels attached to the AD microcontroller converter. Conversion is estimated taking into account both maximum solar irradiance (W/m^2^) and sensor sensitivity.

**Figure 6:**
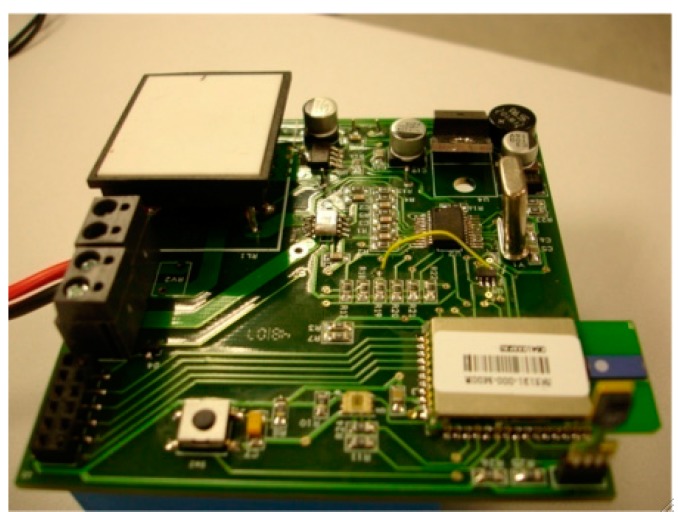
Additional sensor node. AC-voltage and current measurements.

A prototype sensor node designed to measure and collect AC current and voltage values has also been included in the proposed solution. This module collects and measures AC electrical data to enable a deep analysis of current and voltage waveforms using a Fourier series component analysis up to the 20th-harmonic. Figure 6 shows this complementary module in charge of collecting AC electrical variables. Voltage and current variables are measured through a MCP3909 from Microchip [47]. This solution is an energy-metering integrated circuit designed to support the IEC 62503 international standard for metering specifications. It also provides Serial Peripheral Interface (SPI) for connection with the NXP-*Jennic* microcontroller. A voltage transformer (toroidal MCFM32/12 from Multicomp) and a current sensor (ACS712 hall-effect current sensor from Allegro Microsystems [44]) are required for signal conditioning purposes. AC voltage and current signals are measured every minute via SPI from the MPC3909 analogic-to-digital converters during 100 ms of the sampling period: 128 samples per cycle, 50 Hz, 20 ms time period. The data is processed using the Fast Fourier Transform (FFT) algorithm programmed in this node.

### 4.3. Energy Harvesting Module

**Figure 7:**
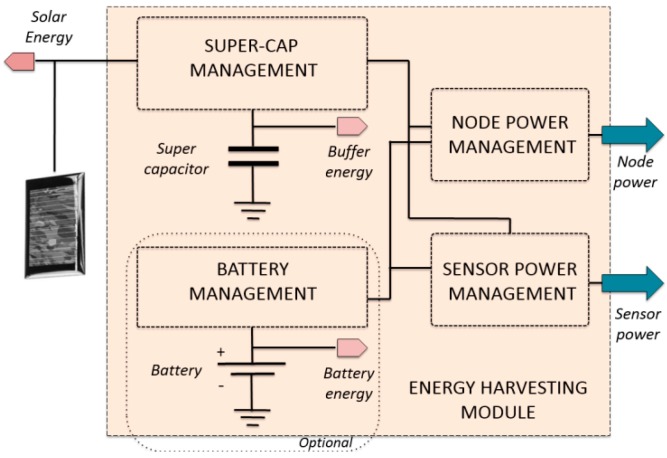
Energy harvesting circuit block diagram.

An additional module has been designed to provide an alternative and autonomous solution as a power supply for the developed nodes. Consequently, nodes are independent for the PV module power generation and data is collected autonomously from the PV plant. There are commercial solutions supplying the developed nodes from solar cells, in accordance with both size and supply constraints. As a result, we selected a solar panel rated at 4 V open-circuit and 3.5-peak voltage, 48.5 mA short-circuit and 45 mA peak current [48] (namely, a mono-crystalline 54 × 43 mm blue solar cell with 15% efficiency and costing $1.6). This low price is very attractive when deploying large wireless sensor networks with advanced energy harvesting. The energy-harvesting module has been designed with a duality on its energy buffering: super-capacitors and batteries, see Figure 7. In comparison with rechargeable batteries, super-capacitors have more charge-discharge cycles (virtually infinite [49]). Moreover, super-capacitors do not suffer partial charge-discharge problems, while batteries may present a discontinuous recharge process. In contrast, capacitor storage is lower than batteries and not enough to guarantee the active mode requirements of microcontrollers (in transmission and reception) in low-solar radiation scenarios. A mixed solution has been thus adapted to cover any possible scenario. The inclusion of this energy-harvesting module enables the nodes to send information at night to enhance safety. However, the node night sleeping time is adjusted as a function of the voltage level of the super-capacitor. Further information on this module can be found in [50].

## 5. Results

### 5.1. Preliminaries

**Figure 8:**
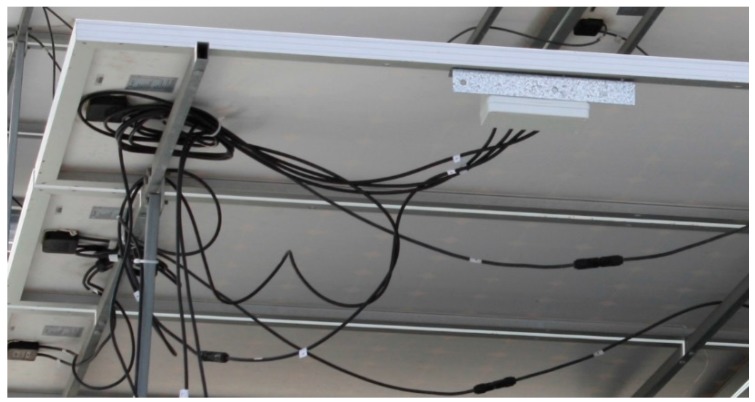
Example of application: sensor nodes on PV modules.

The proposed solution has been assessed from a 93 kWp PV installation connected to the grid and located in south-east Spain. The PV installation has two 40 kW power rate inverters, combined and fed to the distribution power system through a 100 kVA transformer. Each inverter is wired to 30 PV module arrays, and each array consists of 10 strings of series-connected Si-based modules (PhotowattPW-1650). The developed sensor nodes have been connected to a set of PV modules with the aim of collecting both DC-voltage and current values, see Figure 8. An additional sensor node has also been used to measure and collect environmental variables: solar radiation and temperature. From the implemented SSG function, these variables enable the sensor nodes to estimate the *I*-*V* curve for the PV modules. This supposes a relevant characteristic of our proposal: minimizing computational time cost and hardware requirements, and avoiding convergence problems and implicit functions. Table 1 summarizes the corresponding estimated SSG model parameters for the implemented PV module, according to values provided by manufacturers corresponding to open-circuit voltage, short-circuit current and MPP values for a set of G & T parameters [51]. The coefficients of temperature to estimate *I*-*V* curves at different temperature levels are the following [52], expressed in (%/°C) with respect to the Standard Conditions, $\text{α}\left(I_{sc}\right)=0.034$, $\text{β}\left(V_{oc}\right)=-0.36$, δ(MPP)=−0.43. Standard Error (SE), Residual Standard Error (RSE), and MPP Error have been also included in this table as a measure of the tolerance of the estimations.

**Table 1:** Estimated Symmetrized Shifted Gompertz (SSG) Model Parameters.

G (W/m^2^)	T (°C)	k	SE (k)	γ	SE (γ)	b	SE (b)	RSE	MPP Error (%)
**100**	**25**	0.17	0.0024	1.17	0.0009	20.35	1.70	0.0015	−0.73
**200**	**25**	0.34	0.0049	1.19	0.0010	17.09	1.47	0.0031	−0.22
**400**	**25**	0.67	0.0101	1.22	0.0013	14.73	1.36	0.0064	0.13
**500**	**25**	0.84	0.0129	1.23	0.0013	14.11	1.34	0.0082	0.21
**600**	**25**	1.01	0.0157	1.23	0.0014	13.65	1.35	0.0100	0.24
**800**	**25**	1.35	0.0217	1.25	0.0015	13.00	1.38	0.0138	0.25
**1000**	**25**	1.68	0.0281	1.25	0.0016	12.57	1.44	0.0179	0.18
**100**	**45**	0.17	0.0023	1.08	0.0008	20.08	1.53	0.0015	−0.74
**200**	**45**	0.34	0.0047	1.10	0.0010	16.67	1.31	0.0030	−0.19
**400**	**45**	0.68	0.0097	1.13	0.0012	14.26	1.19	0.0062	0.14
**500**	**45**	0.85	0.0123	1.14	0.0013	13.64	1.18	0.0078	0.20
**600**	**45**	1.02	0.0150	1.15	0.0014	13.18	1.17	0.0095	0.22
**800**	**45**	1.36	0.0207	1.16	0.0015	12.53	1.20	0.0132	0.18
**1000**	**45**	1.70	0.0268	1.17	0.0016	12.10	1.24	0.0171	0.08
**100**	**60**	0.17	0.0022	1.01	0.0008	19.89	1.42	0.0014	−0.71
**200**	**60**	0.34	0.0046	1.04	0.0010	16.37	1.20	0.0029	−0.15
**400**	**60**	0.68	0.0094	1.07	0.0012	13.93	1.09	0.0060	0.16
**500**	**60**	0.85	0.0119	1.08	0.0013	13.30	1.07	0.0076	0.19
**600**	**60**	1.02	0.0146	1.09	0.0014	12.84	1.07	0.0092	0.19
**800**	**60**	1.36	0.0201	1.10	0.0015	12.20	1.08	0.0127	0.12
**1000**	**60**	1.70	0.0260	1.11	0.0016	11.77	1.12	0.0165	−0.03

### 5.2. Wireless Network Capacity Analysis

This section analyzes communication between sensor nodes and sink nodes. Sensor nodes are connected to the PV modules through fixed structures. Nodes can be thus grouped into clusters with a central sink, as shown in [17]. In the corresponding field-tests, nodes were deployed in clusters of 20 panels and one meteorological node. However, the solution presented in [17] uses polling, and a periodic data request is sent from the sink to each node. The most adequate solution in the efficiency-simplicity ratio has been adapted to our experiments. Most energy consumption is demanded by radio interfaces and polling increases, involving both transmission and reception time. Alternatively, our sink nodes incorporate an asynchronous wake-up circuit based on RFID [37]. Consequently, a sink node is in charge of sleeping and waking when the sensor node begins a transmission.

Sensor nodes send information by following regular intervals as defined by the sample frequency. Asynchronous transmission schedules implemented at the API level are classified into two groups: (i) those that do not account for other nodes in the cluster and where programming is developed by considering only monitoring requirements; and (ii) those that are aware of the overall cluster size and pay-load requirements. Taking into account the second group (ii), different payload sizes were tested to determine their effect on frame-losses. Each node sends packages of *k*-frames with acknowledgment and a set maximum of re-transmission attempts (*MAX_rec_*). It is a contention-based MAC protocol based on hardware Clear Channel Assessment (CCA) with a random back-off timer. *MAX_rec_* can be also dependent on the node instant-available energy with a 1.1 V threshold, being *T_tx_node_* the time interval taken by the node to transmit a package per cycle, *T_sys_* the Cluster Cycle Time ($\sum_{1}^{n}T_{tx\_node_{i}}$) and *j* a natural number. Each node is then set a sleep mode per cycle according to the expression:

(5) $$T_{sleep}=\left[T_{sys}-T_{tx\_node}\right]+j\cdot T_{sys}$$

**Figure 9:**
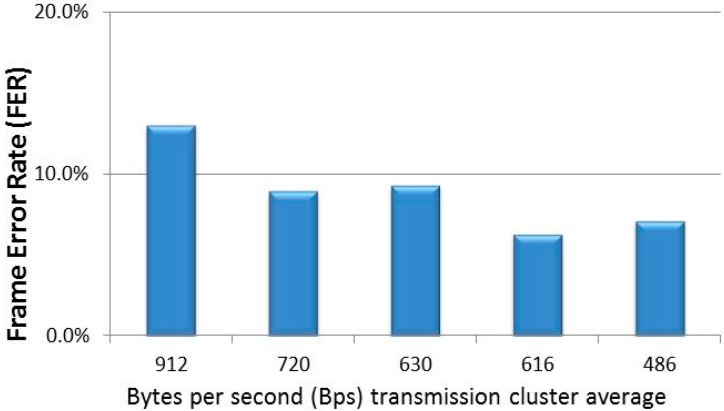
Frame Error Rate (FER) in the experimental scenarios.

**Figure 10:**
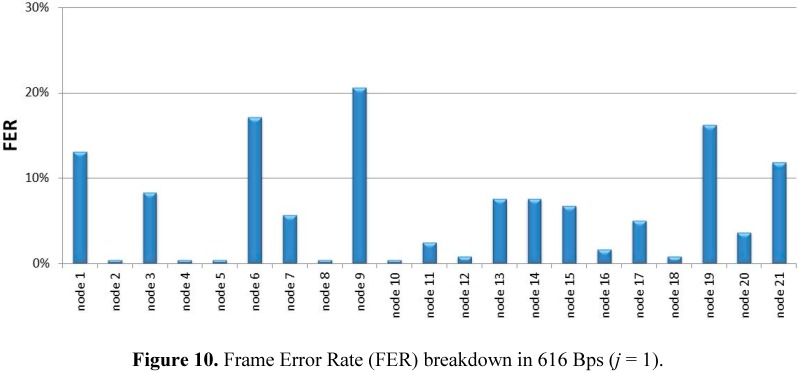
Frame Error Rate (FER) breakdown in 616 Bps (*j* = 1).

Reliability accounts for the ratio of correctly received and transmitted frames. Figure 9 shows the Frame Error Rate (FER) corresponding to five experimental scenarios. The cluster consists of 21 sensor nodes with *j* [0…2]and *MAX_rec_* = 2. The horizontal axis shows the average number of bytes per second (Bps) transmitted by the 21 sensor nodes (Bps excludes ACK load and retransmissions). From the results, FER is not only conditioned by Bps in the cluster, but also by *T_sleep_* heterogeneity. For example, sensor nodes in scenario 2 (720 Bps) and scenario 4 (616 Bps) were all configured with *j* = 1, while sensor nodes in scenario 1 (912 Bps), scenario 3 (630 Bps), and scenario 4 (486 Bps) presented a variability of *j* = [0…2]. Moreover, FER is also conditioned by this range. Nodes were deployed in the PV area depending on the variability of distances to the sink node and the PV-module layout. Figure 10 shows the FER percentage breakdown per node at 616 Bps.

**Figure 11:**
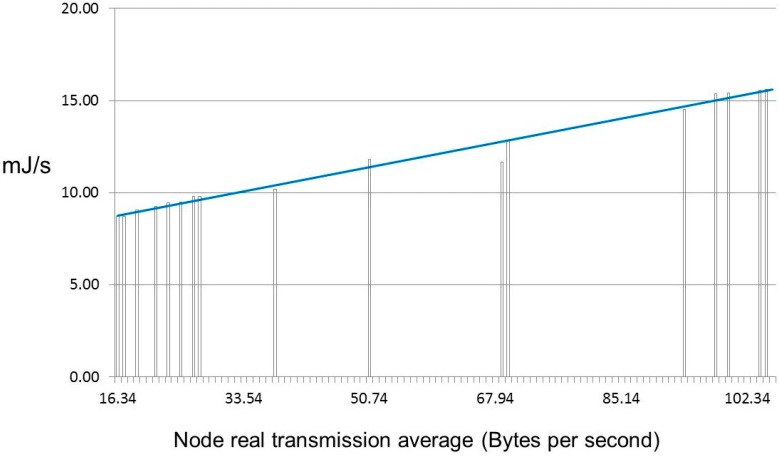
Energy consumption in 630 Bps (*j* = [0…2]).

Power consumption is another relevant parameter useful to characterize the proposed system. Measurements were made taking into account the communication effort, which is always an unquestionable variable in the final power consumption of sensor networks. This type of analysis is thus necessary for cluster size decisions. Although the power supply of the sensor nodes can be obtained from the PV-modules, these nodes also include an additional energy harvesting module that is independent of PV-module performance. Figure 11 shows the energy consumption in mJ per second obtained in scenario 3 (630 Bps). The Pearson product-moment correlation coefficient of this sample ρ*_X,Y_* was 0.986. A value close to 1 implies a linear equation describing the relationship between the average of frames transmitted per second including re-transmitted frames and power demanded per second. Moreover, a sink node with 314 Bps in our sample consumed 34.63 mJ per second.

**Figure 12:**
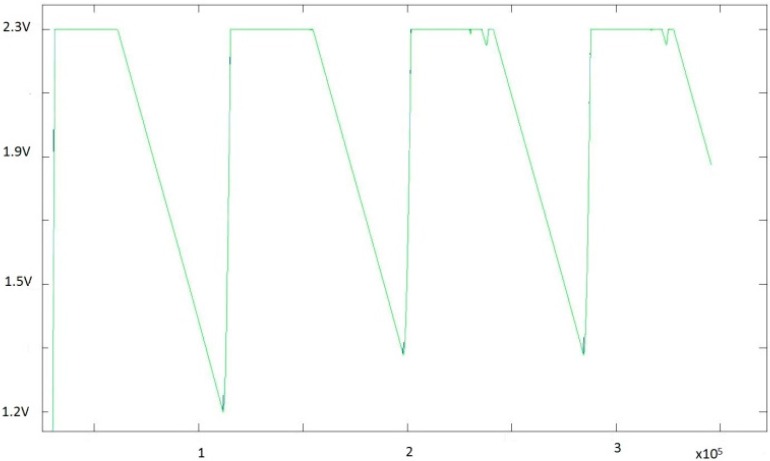
Energy demanded by the capacitor in terms of voltage level (3-days test).

Capacitor charge and discharge periods were observed in a node with the following parameters: 36.2 mA in transmission, 19.7 mA in reception, 5 mA in active, and additional sensor power consumption in active of 6 mA. Transmission time per frame was set to 570 µs and maximum reception time 100 ms with the number of frames per package (*k*) = 15. Additionally, the node active time was 40 ms before transmission, and sleep time after reception was 60 s. Figure 12 shows a three-day test. During daylight, the super-capacitor charge remained within acceptable values. However, at night there was no recharge period, and the super-capacitor charge could only decrement cycle by cycle. Strategies to reduce power consumption at night were based on Energy Neutral Operation (ENO), where sleep time was adjusted as a function of the available energy that could be applied and recommended for PV monitoring [50].

### 5.3. Collected Data and PV Module Performance Estimations

**Figure 13:**
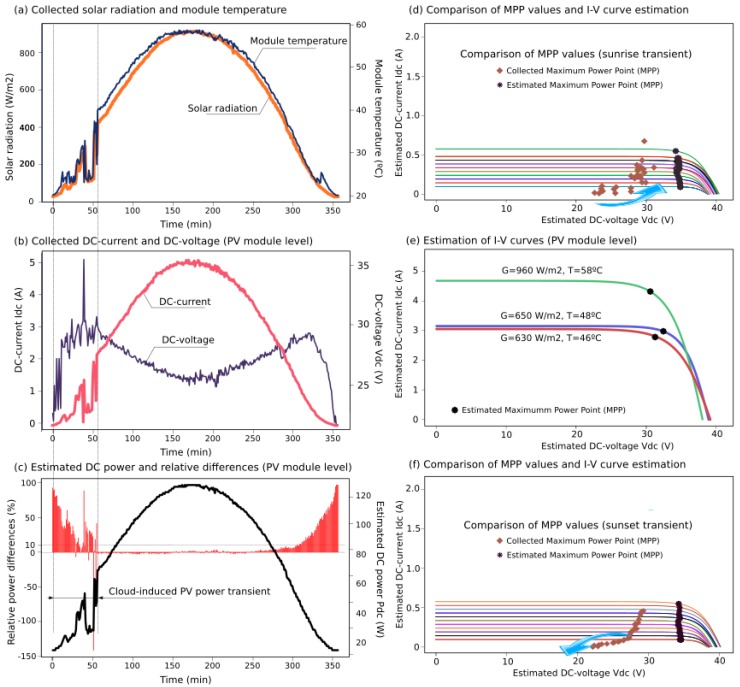
Case Study I: collected data (**a**,**b**); estimations of DC power (**c**); and *I*-*V* curves at the PV module level (**d**–**f**).

**Figure 14:**
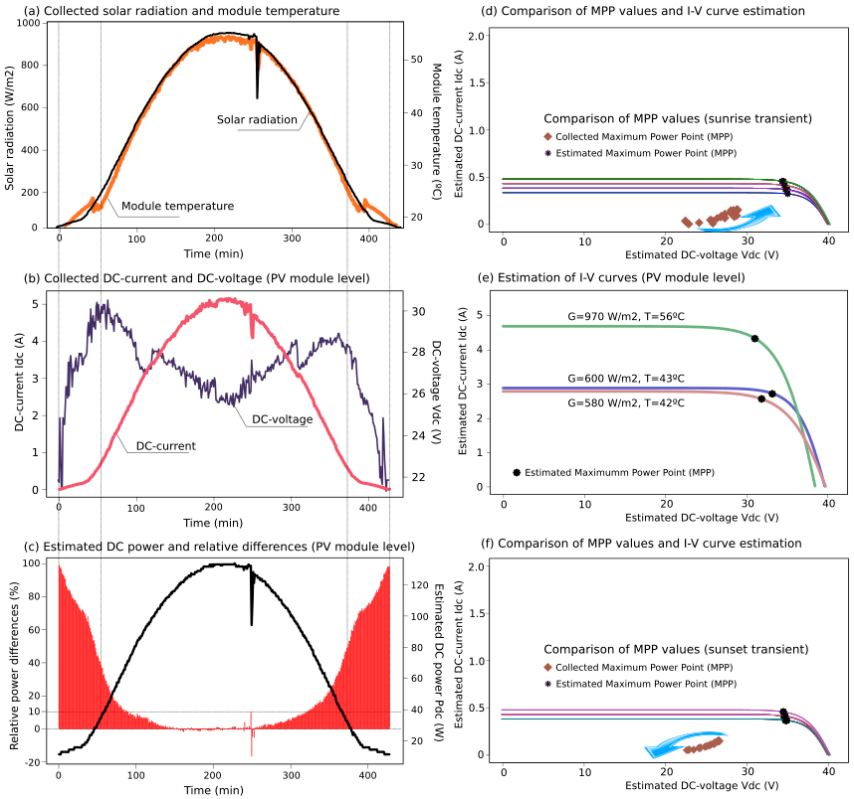
Case Study II: collected data (**a**,**b**); estimations of DC power (**c**), and *I*-*V* curves at the PV module level (**d**–**f**).

Based on the described PV installation, electrical and environmental data was collected by the sensor nodes taking a 2-min sample-time. Additionally, DC values were estimated by the proposed SSG model, determining the theoretical expected power supplied by the PV modules according to the manufacturer data-sheets. Moreover, it was possible to estimate *I*-*V* curves and Maximum Power Point (MPP) values based on the measured G & T variables. Figure 13 and Figure 14 show examples of two days from the collected data, estimations of generated active power, expected *I*-*V* curves and MPP values for the collected G & T data, as well as the relative differences between collected and estimated power values,

(6) $$\left(P_{SSG}-P_{measured}\right)/P_{SSG}$$

To illustrate the proposed solution, various G&T profiles were selected, including oscillations during cloudy days that involved major differences between estimated and collected data, see Figure 13 and Figure 14. According to [53], PV power generation is volatile because cloud-cover produces erratic variations in solar irradiance and PV power production, also affected by the inverter performance and the MPP Tracking algorithm. A comparison between estimated *I*-*V* curves with their corresponding MMP values and collected MPP data are also included for sustained transients as well, such as at sunrise and sunset. Indeed, these transients were observed at 10–21 percent of output over 15 min in previous studies [54]. Nevertheless, all estimated values were obtained with minimal hardware requirements and low computational time costs, avoiding convergence problems and iterative processes. Averaged differences between estimated and collected PV power are below 10%. These differences are higher under the presence of significant solar radiation oscillations and at low levels of irradiance (sunrise and sunset), when it is difficult for the inverters to find the optimal value for the maximum power point [16].

The proposed SSG PV model can be also applied to check the range of electrical power generation, according to the expected data-sheet values provided by the manufacturers. Therefore, a comparison between expected data and collected measurements gives us a preliminary estimation of each PV module performance, avoiding the necessity of removing and analyzing the PV modules in a laboratory environment. In fact, it is a non-practical solution when a large amount of PV modules are to be checked. This efficiency and performance comparison can be used for other aims, such as preventive maintenance analysis, study of efficiency variations and/or parameter modifications along the years. As was discussed in Section 2 [33], affirms that it is necessary to include not only MPP variations, but also the decreasing presented by average module short-circuit current for studies of PV module performance variations over several years. For this reason, the inclusion of the entire estimation of the *I*-*V* curve presents relevant advantages in comparison with only estimating MPP values, and thus providing extensive information to be used in different aspects.

## 6. Conclusions

An innovative solution for monitoring and diagnosing PV solar module performances has been described and assessed in this paper. The proposed system is based on a decentralized WSN at the PV module-level. Low-cost sensor nodes integrate a simple and accurate PV module modeling based on a Shifted Symmetrized Gompertz (SSG) function previously developed and assessed by the authors. The proposed solution has been implemented in a Spanish 93 kWp PV power plant. The system involves four sink nodes and 21 sensor nodes collecting PV module electrical data as well as solar radiation and module temperature. Both DC data and generated power values were estimated by the sensor nodes based on the simple and accurate SSG model. The decentralized system was evaluated and showed a remarkable performance of over 95% in terms of transmission success per node. In addition, an energy-harvesting module was developed for power supply to the nodes, offering autonomous and independent characteristics to the nodes, and including low frequency night transmission to optimize the energy requirements. Field-tests were carried out for several months without additional wiring, and the proposed solution was deemed suitable.
